# Unfolding the Tapestry of Blood Pressure Variability: Revealing Hidden Dimensions

**DOI:** 10.31662/jmaj.2025-0209

**Published:** 2025-06-27

**Authors:** Atsushi Mizuno

**Affiliations:** 1 Department of Cardiovascular Medicine, St. Luke’s International Hospital, Tokyo, Japan

**Keywords:** blood pressure variability, heart failure with preserved ejection fraction, temporal dynamics, autonomic dysfunction, visit-to-visit variability, higher-order variability

Blood pressure variability (BPV) has consistently been associated with the development and prognosis of cardiovascular diseases ^(1)^. One of the major mechanisms underlying this relationship is autonomic dysfunction. BPV reflects an imbalance between the sympathetic and parasympathetic nervous systems, with decreased heart rate variability strongly linked to blood pressure (BP) instability and increased cardiovascular risk. Furthermore, patterns such as non-dipping nocturnal BP and morning surge are thought to result from impaired autonomic regulation. Beyond autonomic factors, other mechanisms contributing to increased BPV include arterial stiffness, abnormal volume regulation by the kidneys, hormonal fluctuations (such as activation of the renin-angiotensin-aldosterone system), and systemic inflammation and oxidative stress. These factors interact in a complex manner, reinforcing each other to amplify BPV and, thereby, worsen cardiovascular risk.

BPV spans a wide range of temporal scales, from instantaneous to long-term changes. As illustrated in Figure 1, beat-by-beat variability captures fluctuations occurring with each heartbeat; minutes/postural variability reflects BP responses to postural changes; diurnal variability characterizes fluctuations across daytime and nighttime; day-by-day variability addresses daily shifts due to behavioral factors; visit-to-visit variability examines changes across clinic visits spaced months apart; seasonal variability arises from environmental and physiological changes across seasons; and yearly variability encompasses the effects of aging and disease progression.

**Figure 1.**
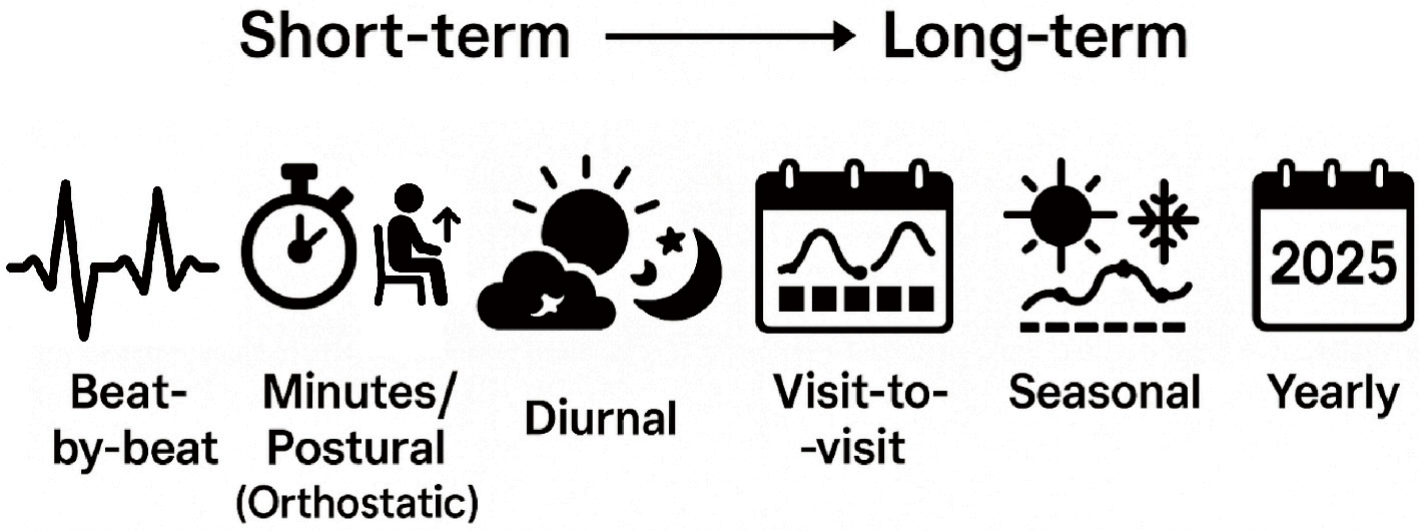
Temporal Spectrum of Blood Pressure Variability. Blood pressure variability (BPV) occurs across a wide range of timescales. This figure illustrates key categories from short- to long-term: beat-by-beat, minutes/postural (orthostatic), diurnal, visit-to-visit, seasonal, and yearly variability. Understanding BPV across these dimensions is essential for capturing its full clinical significance.

From a physical and natural science perspective, when multiple rhythms across different timescales interact, they produce complex dynamic patterns―such as beat phenomena, moiré interference, and nonlinear chaos―that cannot be explained by a single periodicity. Similarly, in BPV, short-term fluctuations (e.g., within a day) and long-term changes (e.g., seasonal or annual trends) overlap and interact, making it insufficient to evaluate cardiovascular risk using only average BP levels.

Various indices have been developed to assess BPV. These include the maximum-minimum difference, standard deviation (SD), coefficient of variation, variation independent of mean (VIM), average successive variability, average real variability, standard deviation about the regression line, and root mean square of successive differences. All of these metrics fundamentally aim to extract the true variability in BP, adjusting for the confounding effects of the absolute BP level itself (Table 1).

**Table 1.** Summary of Blood Pressure Variability Metrics: Calculation Methods, Units, and Key Characteristics.

Metric name	Calculation method	Unit	Key characteristics
Max-Min Difference	Maximum BP value minus minimum BP value over a period	mmHg	Simple measure of range; sensitive to extreme values
Standard Deviation (SD)	Square root of the average squared deviation from the mean	mmHg	Captures overall dispersion; influenced by mean BP level
Coefficient of Variation (CV)	SD divided by mean BP, multiplied by 100	%	Standardizes variability relative to mean BP; expressed as a percentage
Variation Independent of Mean (VIM)	SD divided by mean BP to the power x, multiplied by population mean to the power x	(mmHg)	Assesses variability independently of mean BP; adjusts for baseline BP differences
Average Successive Variability / Average Real Variability (ARV)	Mean of the absolute differences between successive BP measurements	mmHg	Reflects short-term or visit-to-visit fluctuations between consecutive readings; ARV term often used in clinical contexts to reduce overestimation from outliers
Standard Deviation about Regression Line (SDreg)	SD of the residuals from the regression line of BP over time	mmHg	Measures variability around long-term BP trends; removes influence of systematic trends
Root Mean Square of Successive Differences (RMSSD)	Square root of the mean of the squared successive BP differences	mmHg	Captures beat-to-beat or short-term variability; emphasizes rapid fluctuations

BP: blood pressure; Max: maximum; Min, minimum.

The prognostic importance of BPV has been demonstrated not only in normotensive and hypertensive individuals but also in those with established cardiovascular diseases. However, evidence in heart failure with preserved ejection fraction (HFpEF) remains limited. In the TOPCAT trial, analyses using VIM suggested that greater BPV may predict worse outcomes in HFpEF, yet the overall evidence remains inconclusive ^(2)^. In the current study, Komiyama et al. ^(3)^ evaluated the impact of visit-to-visit BPV on mortality and heart failure rehospitalization in a Japanese single-center cohort of HFpEF patients. Although a significant association was observed, similar to prior findings, clear clinical cutoffs and actionable thresholds have not yet been established, highlighting the need for further prospective studies.

From a clinical standpoint, efforts to reduce systolic BPV have focused on lifestyle interventions and pharmacological therapies. Calcium channel blockers, such as amlodipine, have been shown to reduce BPV more effectively compared to beta-blockers. In contrast, medications such as renin-angiotensin system inhibitors and beta-blockers, despite their established benefits for heart failure outcomes, appear less effective in stabilizing BPV. Consequently, no targeted intervention strategies for BPV modulation have yet been firmly established. Recently, interest has grown regarding how newer therapeutic classes―such as sodium-glucose cotransporter 2 inhibitors, and glucagon-like peptide-1 receptor agonists for the heart failure population―may influence BPV. Although direct evidence remains limited, their potential impact on variability, particularly from a cardiovascular-kidney-metabolic perspective, warrants further attention ^(4)^.

Importantly, the field of BPV research is evolving from simple assessments of dispersion (such as SD) toward examining higher-order variability, i.e., how variability itself changes over time. Capturing the temporal evolution of BPV requires sophisticated analytic techniques. Fourier transform enables the decomposition of BP time-series data into constituent frequency components, revealing dominant periodicities such as circadian rhythms. Wavelet transform further allows for simultaneous analysis of time and frequency, enabling the detection of transient phenomena. Poincaré plots, by plotting each BP measurement against the next, visualize short-term variability and pattern stability.

Harnessing these methods, researchers can move beyond static measures of BP variability to dynamic, time-sensitive assessments, potentially unveiling novel prognostic markers and therapeutic targets. As BPV analysis becomes increasingly nuanced, integrating principles of differential calculus―such as examining the first and second derivatives of BP over time―may become essential for a deeper understanding of cardiovascular risk.

In conclusion, adopting these advanced perspectives and analytic approaches will be critical in fully elucidating the complex relationship between BPV and cardiovascular outcomes. In this context, the study by Komiyama et al. ^(3)^ provides valuable new insights by focusing on HFpEF―a population for which evidence regarding BPV and prognosis has been relatively scarce. Moving forward, it will be essential not only to apply these methods within heart failure populations but also to extend their use across the broader spectrum of cardiovascular disease. A more individualized, as well as comprehensive, evaluation of BPV patterns―capturing the multilayered complexity of BP fluctuations―will be required. Leveraging digital technologies for continuous, high-resolution monitoring and analysis will likely play a central role in advancing this field.

## Article Information

### Conflicts of Interest

None

### Acknowledgement

The conceptual figure illustrating blood pressure variability across timescales (Figure 1) was created with the assistance of OpenAI’s ChatGPT, utilizing AI-driven graphic generation support.

### Disclaimer

Atsushi Mizuno is one of the Editors of JMA Journal and on the journal’s Editorial Staff. He was not involved in the editorial evaluation or decision to accept this article for publication at all.
